# What Is the *de-qi*-Related Pattern of BOLD Responses? A Review of Acupuncture Studies in fMRI

**DOI:** 10.1155/2013/297839

**Published:** 2013-02-07

**Authors:** Jinbo Sun, Yuanqiang Zhu, Yang Yang, Lingmin Jin, Karen M. von Deneen, Wei Qin, Jie Tian

**Affiliations:** ^1^Life Sciences Research Center, School of Life Sciences and Technology, Xidian University, Xi'an, Shaanxi 710071, China; ^2^Institute of Automation, Chinese Academy of Sciences, Beijing 100190, China

## Abstract

*de-qi*, comprising mostly subjective sensations during acupuncture, is traditionally considered as a very important component for the possible therapeutic effects of acupuncture. However, the neural correlates of *de-qi* are still unclear. In this paper, we reviewed previous fMRI studies from the viewpoint of the neural responses of *de-qi*. We searched on Pubmed and identified 111 papers. Fourteen studies distinguishing *de-qi* and sharp pain and eight studies with the mixed sensations were included in further discussions. We found that the blood oxygenation level-dependent (BOLD) responses associated with *de-qi* were activation dominated, mainly around cortical areas relevant to the processing of somatosensory or pain signals. More intense and extensive activations were shown for the mixed sensations. Specific activations of sharp pain were also shown. Similar BOLD response patterns between *de-qi* evoked by acupuncture stimulation and *de-qi*-like sensations evoked by deep pain stimulation were shown. We reckon that a standardized method of qualification and quantification of *de-qi*, deeper understanding of grouping strategy of *de-qi* and sharp pain, and making deep pain stimulation as a control, as well as a series of improvements in the statistical method, are crucial factors for revealing the neural correlates of *de-qi* and neural mechanisms of acupuncture.

## 1. Introduction

Acupuncture, as a key component of traditional Chinese medicine (TCM) and an alternative and complementary therapy in western society, has been widely used all over the world. As an old concept that appeared in a very early Chinese classic text, *de-qi *was believed to be an indispensable component of acupuncture treatment [1]. In recent years, the importance of *de-qi* has been investigated based on the modern biological and medical framework [2–14]. Several studies reported the correlation between the *de-qi* sensations and analgesic effects [2–4, 10–12] or the electroencephalogram changes [9], whereas others do not [8, 13, 14]. Therefore, the role of *de-qi* in acupuncture treatment is still controversial [1, 5, 6, 15].

Many researchers have begun to qualify and quantify sensations of *de-qi *[15–27], because they argued that the lack of a competent measure of *de-qi* affected the association between *de-qi* and the clinical outcomes [1, 18, 19]. Most studies reported that *de-qi* sensations involved numbness, heaviness, aching, dull pain, and tingling [1, 16, 18, 19, 23, 25, 27]. Other sensations such as fullness/distention, soreness, and pressure were also included in part of the studies [1, 16, 19, 27]. The dimensions of sensations of *de-qi* were largely different, which ranged from four [19] to seventeen [25]. Although there have not a consensus that which sensations were involved in *de*-*qi*, researchers focused on qualifying and quantifying sensations of *de-qi*, and they agreed that deqi includes specific sensations for acupuncture stimulation [1, 16, 18, 27]. Besides, another consensus was reached that as a conventional component of the needling sensations presented during acupuncture manipulation, sharp pain is noxious and is what acupuncturists try to avoid during needle manipulation [1, 16, 18, 25]. It is often excluded from the components of *de-qi* and regarded as the sensation that is irrelevant to the acupuncture effect [1, 16, 18, 25].

With the aid of functional magnetic resonance imaging (fMRI) techniques, it is possible to explore the neural responses of *de-qi *and further interpret the neural mechanisms of acupuncture. More than one hundred studies have been published to explore the neurobiological mechanisms of acupuncture in the past few decades [28–36]. However, few of them paid close attention to the neural correlation of *de-qi.* Therefore, several fundamental issues about the central responses of *de-qi* remain open to debate. First of all, although researchers agreed that *de-qi* has specific sensations for acupuncture stimulation [16], the sensory quality of *de-qi* was similar to that of deep pain constructed by intramuscular injections of hypertonic saline, which mainly included tenderness, heaviness, aching, cramping, throbbing, and gnawing [25, 37]. Thus, the quality of the sensation of *de-qi* was likely nonspecific [38, 39]. Up to now, however, direct comparisons of blood oxygenation level-dependent (BOLD) responses between *de-qi* sensations evoked by acupuncture stimulation and *de-qi*-like sensations evoked by deep pain stimulation have not yet been investigated. Only one recent study involved the indirect comparisons of these two stimulations, which had a similar sensory quality, in the discussion [40]. Secondly, in the domain of pain studies, it is generally known that differences in the quality and origin between sharp pain and deep pain are remarkable [41]. Abundant evidence from animal investigations [42, 43], clinical data [44–46], and human neuroimage studies [37, 47–52] indicated different brain processing pathways for acute superficial pain (sharp pain) and deep pain. The sharp pain following acupuncture stimulation is sometimes perceived by subjects. However, most previous acupuncture fMRI studies did not explicitly distinguish the needle sensations into *de-qi* and sharp pain. To make matters worse, significant incompatibilities were shown across the results of several studies which excluded subjects who experienced sharp pain [22, 23, 25] or divided subjects into two groups according to whether subjects experienced sharp pain during acupuncture manipulation [33, 53–55].

In this paper, we would like to summarize the results of fMRI-based acupuncture studies from the perspective of the general pattern of central BOLD responses of *de-qi*. We aim to organize the evidence about three fundamental questions. First, what is the pattern of central responses of *de-qi* evoked by during acupuncture stimulation? Second, how are the distinct patterns of central BOLD responses associated with *de-qi* and sharp pain? Thirdly, are the patterns of central BOLD responses associated with *de-qi* specific from those of deep pain in pain studies? In addition, we hope to offer several suggestions for future studies of the neural correlates of *de-qi* evoked by acupuncture stimulation.

## 2. Identification of the Relevant Literature

The acupuncture studies involved in this paper were first identified by searching on Pubmed using the key words “acupuncture” or “electroacupuncture” in the title and “fMRI” or “functional magnetic resonance imaging” in the title/abstract (four combinations of key words: “acupuncture” and “fMRI,” “acupuncture” and “functional magnetic resonance imaging,” “electroacupuncture” and “fMRI,” and “electroacupuncture” and “functional magnetic resonance imaging”). About 111 original studies published in peer-reviewed journals in English were included in the search (two studies in the Evidence-Based Complementary and Alternative Medicine with the state of “in press” were also involved). Firstly, 17 review articles were excluded. Secondly, 23 studies which did not evaluate and record subjects' needling sensations were discarded from further discussions. Thirdly, 13 studies that were based on data-driven methods were excluded. Fourthly, 4 studies that aimed to explore the continuous effect of acupuncture were also excluded. Fifthly, other 15 studies were also not included because of the lack of *de-qi*-related BOLD responses (2 were behavioral studies, 2 were magnetoencephalogram-based studies, 4 studies were associated with the therapeutic/placebo effect of acupuncture, 1 study was laser-acupuncture-based, and 5 studies lacked the whole brain's one-sample *t-*test results). In the remaining 39 studies, 17 studies asked or evaluated the subjects' *de-qi* but did not mention whether subjects underwent sharp pain [36, 38, 56–70]. Eight studies which asked or evaluated the subjects' *de-qi* and sharp pain but did not distinguish *de-qi* and sharp pain in the fMRI analysis [53, 71–77] were named as “mixed pattern studies” and summarized in Table 3. Fourteen studies that excluded subjects who experienced sharp pain for the fMRI data analysis [32, 34, 78–85] or divided subjects into two groups according to whether subjects experienced sharp pain during acupuncture manipulation [33, 40, 54, 55] were named as “pure *de-qi* pattern studies” and are summarized in Table 2. The experimental details and the methodological details of the 14 *de-qi*-related studies are shown in Table 1 and Table 5, respectively.

## 3. General Observations

Reviewing the studies of acupuncture in fMRI, we found that *de-qi* and sharp pain were not distinguished in most studies (97 out of 111, 87%). Therefore, limited resources could be used for summarizing the *de-qi* BOLD response patterns. We suggest that it is necessary to differentiate *de-qi* and sharp pain in further fMRI acupuncture studies. In the following part, we will summarize the BOLD response patterns of pure *de-qi*, the BOLD response patterns of mixed sensations, the similarities/differences of BOLD response patterns between pure *de-qi* and mixed sensations, similarities/differences of BOLD response patterns between pure *de-qi* and *de-qi*-like sensations evoked by deep pain stimulation, and the comparison of *de-qi*-related regions with regions generally activated in acupuncture fMRI studies, which were summarized in other reviews [39, 86].

### 3.1. The BOLD Responses Pattern of Pure *de-qi*

The common activations (frequency  > 4 or 29%) of *de-qi* were the SI, SII, thalamus, MI, cerebellum, insula, inferior parietal lobe, and anterior MCC. The common deactivations of *de-qi* were the ACC, amygdala, hippocampus, parahippocampus, hypothalamus, temporal pole, and PCC. Most of the commonly responding regions (11/15 or 73%) were divergent across studies.

### 3.2. The BOLD Response Patterns of Mixed Sensations

The common activations (frequency  >  2 or 25%) of mixed sensations were the SI, SII, MI, cerebellum, SMA, insula, IMG, DLPFC/VLPFC, and pMCC. The common deactivations of *de-qi* were the STG, inferior temporal gyrus, precuneus, lingual gyrus, occipital gyrus, and PCC.

### 3.3. The Similarities/Differences of BOLD Response Patterns between Pure *de-qi* and Mixed Sensations

In the 14 “pure *de-qi* pattern studies,” only two studies performed the between-group analysis that statistically compared the *de-qi* group and mixed group (*de-qi*  + sharp pain) based on a two-sample unpaired *t*-test [40, 55]. In Hui et al., 2009, significant differences in the posterior cingulate/precuneus, pregenual cingulate/frontal pole, subgenual area, orbitofrontal cortex, temporal pole, amygdala, hippocampus, parahippocampus, hypothalamus, cerebellar vermis (lobules VII and VIII), SII, and anterior middle cingulate were shown between the *de-qi* group and mixed group. Specifically, activity of BOLD signals was significantly less activated or more deactivated in the *de-qi* group than that in the mixed group. In Sun et al., 2012, significantly stronger activations of the *mixed* group were presented in the bilateral putamen, the bilateral thalamus, and the bilateral cerebellum (CrusI and CrusII) [40]. In these regions, BOLD signals of the *de-qi* group were barely changed, while significant BOLD responses were shown in the mixed group.

### 3.4. The Similarities/Differences of BOLD Response Patterns between Pure *de-qi* and *de-qi*-Like Sensations Evoked by Deep Pain Stimulation

In contrast to superficial (cutaneous) pain, deep pain (originating from muscle, joints or viscera) is dull, diffuse, and difficult to localize [37, 50]. In Henderson et al., 2006, the researchers used intramuscular injections of hypertonic saline to construct a deep pain model [37]. The subjective sensations under this deep pain mainly included tenderness, heaviness, aching, cramping, throbbing, and gnawing, which were similar to that of *de-qi* evoked by acupuncture stimulation.  Table 4 summarizes the activations and deactivations from the deep pain studies [37, 48, 50, 87, 88]. Common activations (frequency >  2 or 40%) were seen in the anterior cingulate, posterior cingulate, SI, SII, MI, insula, cerebellar cortices, inferior parietal, claustrum, and thalamus. A common deactivation was in the perigenual cingulate. Most of the activations for deep pain were consistent with that of *de-qi* evoked by acupuncture stimulation, except for the anterior cingulate and posterior cingulate, which were commonly deactivated during *de-qi*. Besides, the amygdala, hippocampus, parahippocampus, hypothalamus, and temporal pole were deactivated for *de-qi* but not for deep pain.

### 3.5. The Comparison of *de-qi*-Related Regions with Regions Generally Activated in Acupuncture fMRI Studies

Huang et al., 2012, summarized the general BOLD responses in acupuncture fMRI studies [86]. The supramarginal gyrus/insula/SII, presupplementary motor area/middle cingulate, thalamus, and precentral gyrus were the most commonly found activations. Besides, the anterior cingulate, subgenual cortex, amygdala/hippocampal formation, ventromedial prefrontal cortex, and posterior cingulate were the most common deactivations. Beissner, 2011, summarized the common BOLD responses evoked by acupuncture stimulation based on studies which met the methodological inclusion criteria [39]. The most frequently activated cortical areas were the SII, insula, SI, cerebellum, thalamus, MI, STG, visual cortices, IFG, SMA/pre-SMA, basal ganglia, MTG, and ACC. Significant differences in the regions generally activated in acupuncture fMRI studies were shown between these two previous reviews. When comparing our results with theirs, we found that our results were more similar to the results of Huang et al., 2012, but were more extensive (particularly showing more deactivations) than the results of Beissner, 2011. We argued that it was because our review of *de-qi*-related patterns and results of Huang et al., 2012, lacked the methodological inclusion criteria [39]. 

## 4. Discussion

This paper summarized the results of fMRI-based acupuncture studies from the perspective of the general pattern of central BOLD responses of *de-qi*. Three fundamental issues are discussed later. In addition, several suggestions for future studies of the neural correlates of *de-qi* evoked by acupuncture stimulation were offered.

### 4.1. Activation-Dominated BOLD Responses Associated with *de-qi* during Acupuncture Stimulation

The most robust BOLD responses evoked by acupuncture stimulation were in the SII, insula, SI, cerebellum, thalamus, MI, STG, visual cortices, IFG, SMA/pre-SMA, basal ganglia, MTG, and ACC. Under the methodological inclusion criteria [39], only the deactivation of the occipital cortex was shown. For the *de-qi-*related BOLD responses, a similar activation pattern was shown, including in the SI, SII, thalamus, MI, cerebellum, insula, inferior parietal lobe, and anterior MCC. Besides, several deactivations were seemingly the common pattern of *de-qi*. However, we argued that the reliability of these deactivations was poor. We inferred that several possible reasons might contribute to these deactivation patterns. Firstly, the average of the repeated runs for each subject was applied. Repetition may itself alter the distribution of the activated regions due to the influence of memory and expectation [48]. Secondly, global normalization, a questionable data processing step adopted in several fMRI-based acupuncture studies, can introduce an artificially negative relationship with the task [83, 89–92]. Particularly, our recent study clarified that for the fMRI-based acupuncture data, global normalization significantly changed the activation-dominated results to deactivation-dominated ones [83]. Therefore, we suggested that BOLD responses associated with *de-qi* during acupuncture stimulation should be activation dominated.

### 4.2. Distinct Patterns of Central BOLD Responses Associated with *de-qi* and Sharp Pain

Since subjects with pure sharp pain during acupuncture stimulation were difficult to obtain, the differences between *de-qi* and sharp pain evoked by acupuncture stimulation could not be presented directly. Therefore, we had to infer their differences between the *de-qi* and *mixed* sensations. However, studies which were statistically compared with the BOLD responses between *de-qi* and mixed sensations were also a scarcity [40, 55]. Our recent study focused on these issues and indicated that both the quantitative and qualitative differences of BOLD responses between *de-qi* and mixed sensations evoked by acupuncture stimulation were distinct [40]. We inferred that the pattern of BOLD responses for sharp pain might be partly separated from that of *de-qi* in the spatial distribution. The subjects with sharp pain should be excluded from those with only *de-qi* when exploring the central BOLD responses during acupuncture stimulation [40].

### 4.3. Similar BOLD Response Patterns between *de-qi* Evoked by Acupuncture Stimulation and *de-qi*-Like Sensations Evoked by Deep Pain Stimulation

Most common activations of *de-qi* in this paper were shown in the aforementioned deep pain studies, which might indicate similar central processing of the same origin of stimulation and similar subjective sensations. Due to individual variability of brain morphology and differences in experimental design, the central patterns of activation during deep pain and acupuncture stimulation were difficult to compare between studies. Therefore, we suggested that a specific central effect of *de-qi* during acupuncture stimulation might be illustrated after comparing it directly to deep pain stimulation.

### 4.4. Several Suggestions for Future Studies of the Neural Correlates of *de-qi* Evoked by Acupuncture Stimulation

First of all, for better understanding what *de-qi* is and exploring its possible role in acupuncture, many researchers have been engaged in qualifying and quantifying *de-qi* [1, 15–27]. However, in the aforesaid fMRI-based acupuncture studies about *de-q*i, different sensation questionnaires were used to quantify subjects' acupuncture sensations (see Table 1 for details). This may lead to two results. One is that the kinds of acupuncture sensations recorded by different studies are partially different. The other is that the same score of an acupuncture sensation between different studies refers to a different subjective intensity experienced by subjects due to the various definitions of the same score between different studies. Thus, acupuncture sensations recorded by different studies are inappropriate to compare. We suggest that further studies should pay more attention to the quantification questionnaire of *de-qi* and try to apply a standardized quantification method to better control the experimental conditions [38, 76].

Secondly, another important discrepancy among studies is the definition of grouping. In Hui et al. and Asghar et al. [33, 53], they both grouped subjects into two groups (the *de-qi* group and mixed group). But the definition of the “*de-qi* group” was totally different in the two studies. In the studies of Hui, the *de-qi* group referred to subjects who only experienced *de-qi* during acupuncture, while in Asghar et al., the *de-qi* group referred to subjects whose *de-qi* scores were greater than sharp pain, which were similar to the Sun et al.'s sharp pain group (de-qi sensation mixed with sharp pain). Sun et al.'s study indicated that even a little sharp pain mixed in, both the quantitative and qualitative differences of BOLD responses between de-qi and mixed sensations evoked by acupuncture stimulation were significant [40]. Therefore, we proposed that subjects with sharp pain should be separated from those with only *de-qi* when exploring the central BOLD responses during acupuncture stimulation. Further comparisons between *de-qi* and sharp pain should be accumulated. Besides, the deep pain stimulations, which had a similar sensory quality to *de-qi* evoked by acupuncture stimulation, could serve as a valid control for acupuncture.

At last, methodological problems and differences may partly contribute to the discrepancies among studies which were reviewed here. Beissner and Henke found that most acupuncture studies lacked adequate strict statistical methods and suggested that several methodological problems should be solved to facilitate acupuncture studies [93]. The improvements for obtaining more reliable results included a larger sample size, corrected threshold, and a more robust method of statistical inference [94]. With regards to the studies reviewed in this paper, an inappropriate group analysis method, a too small sample size, and the liberal threshold applied by the studies could be the most important problems (see Table 3 for details). For instance, first, Hui et al. [33] applied the fixed-effect model in the group analysis, which could produce positive group results even when only a single subject has strong activations [93]. Second, Hui et al. recruited 13 subjects in their study, and only two subjects experienced the mixed sensation [54]. Hui et al. recruited 15 subjects and a mixed group with 4 subjects [33]. Such a sample size (2 subjects and 4 subjects) is too small to obtain group results with sufficient statistical power. Third, several studies adopted global normalization [33, 53, 55], which is a questionable data processing step and can introduce an artificially negative relationship with the task [89–92, 95–98]. Particularly, our recent study clarified that for the fMRI-based acupuncture data, global normalization significantly changed the activation-dominated results to deactivation-dominated ones [83]. As a whole, solving these crucial methodological problems could greatly help studies on neural correlates of *de-qi* to obtain more repeatable and reliable results. Thus, we suggest that future studies should focus on improvements in their methodological problems which we believe could shed light on studies of neural correlates of *de-qi*. 

## 5. Synopsis and Possible Solutions

*de-qi*, comprising mostly of subjective sensations during acupuncture, is traditionally considered as a very important component of the possible therapeutic effects of acupuncture. Thus, it is of great importance to reveal the neural correlates of *de-qi* which may benefit the understanding of neural mechanisms of acupuncture. However, previous studies in fMRI involving the BOLD response of* de-qi* were limited and did not reach consistent conclusions on the neural response pattern of *de-qi *in the brain. In this paper, we summarized previous fMRI studies on the neural responses of *de-qi *and answered three fundamental questions. For the first question concrning the pattern of the central responses of *de-qi* evoked by acupuncture stimulation, our answer was that the BOLD responses associated with *de-qi* during acupuncture stimulation were activation dominated, mainly around cortical areas relevant to the processing of somatosensory or pain signals. For the second question on how the distinct patterns of central BOLD responses are associated with *de-qi* and sharp pain, our answer was that more intensive and extensive activations were shown for the mixed sensations evoked by acupuncture stimulation. Specific activations of sharp pain were also shown. For the third question asking if the patterns of central BOLD responses were associated with *de-qi* specifically from those of deep pain in pain studies, our answer was that similar BOLD response patterns between *de-qi* evoked by acupuncture stimulation and *de-qi*-like sensations evoked by deep pain stimulation were shown. Finally, we reckon that a standardized method of qualification and quantification of *de-qi*, a deeper understanding of grouping strategy of *de-qi* and sharp pain, and marking the deep pain stimulation as a control, as well as a series of improvements in the statistical method, are crucial factors for revealing the neural correlates of *de-qi* and neural mechanisms of acupuncture. 

## Figures and Tables

**Table 1:** Experimental details of fMRI studies on BOLD responses of *de-qi*.

Studies	Acupoints used	Stimulation	Sensations evaluated	Grouping
Studies that compared central BOLD responses between *de*-*qi* and sharp pain groups				

Hui et al., 2000 [54]	LI4	Manual	*de-qi*, pain	*de-qi* group: 11; sharp pain group: 2
Hui et al., 2005 [33]	ST36	Manual	Their own scale	*de-qi* group: 11; sharp pain group: 4
Hui et al., 2009 [55]	LI4, LV3, ST36	Manual	Their own scale	*de-qi* group: 37 (scans); sharp pain group: 29 (scans)
Sun et al., 2012 [40]	ST36	Manual	Their own scale	*de-qi* group: 19; sharp pain group: 19

Studies where no subjects reported sharp pain/excluded subjects who experienced sharp pain from fMRI analysis				

Napadow et al., 2005 [34]	ST36	Electro	Their own scale	Total: 13
Jeun et al., 2005 [78]	GB34	Manual	Their own scale	Total: 10
Kong et al., 2007 [79]	GB37, UB60	Electro	SASS	Total: 8
Zhou and Jia, 2008 [66]	HT7, ST36, ST40, KI3	Electro	Their own scale	Total: 26
Fang et al., 2009 [32]	LV3, LV2, ST44	Manual	Their own scale	Total: 10
Na et al., 2009 [80]	GB34	Manual	Their own scale	Total: 12
Bai et al., 2010 [81]	PC6, PC7, GB37	Electro	Their own scale	Total: 36
Qiu et al., 2010 [82]	LI4, LV3, ST36	Manual	Their own scale	Total: 38
Fang et al., 2012 [85]	CV4, CV12	Electro	Their own scale	Total: 17
Sun et al., 2012 [40]	ST36	Manual	Their own scale	Total: 52

**Table 2:** *de-qi-*related BOLD responses evoked by acupuncture stimulation.

Regions	(Reference no.)	
	Activations	Deactivations
Somatosensory system		
SI	[32, 34, 40, 54, 79, 84]	[79, 82]
SII	[32–34, 54, 55, 79, 85]	
Thalamus	[32, 34, 40, 80, 83–85]	[33]
Caudate	[80]	[33, 54]
Putamen	[34, 40, 80]	[54]
Brainstem	[33]	[33, 34]
Motor system		
M1	[40, 78, 78, 80, 83]	[33]
Cerebellum	[40, 79–81, 83, 84]	[33, 55, 82]
SMA	[32, 85]	
Sensory integration system		
Insula	[32, 34, 40, 79, 81–85]	[33, 54]
ACC		[33, 54, 55, 85]
Special senses		
IFG	[40, 83]	
STG	[40, 83, 84]	
MTG		
Frontal cortex		
vmPFC	[82]	[33, 34, 85]
Frontal Pole	[34]	[32, 33, 55]
Superior frontal gyrus	[80]	[79]
Medial orbital prefrontal cortex		[79]
Medial frontal gyrus		[79]
Ventromedial frontal pole		[34]
Dorsomedial frontal pole		[32, 34]
DLPFC/VLPFC	[34]	
DMPFC	[34]	
OFC/MPFC	[34]	[82]
Paracentral lobule	[33]	[79]
Pregenual cingulate cortex		[82]
Subcortical gray matter		
N. accumbens/septi		[33, 54]
Amygdala	[81]	[32–34, 54, 55, 82]
Hippocampus	[84]	[32, 33, 54, 55, 82]
Parahippocampus	[34]	[32, 33, 54, 55, 79, 82]
Hypothalamus	[79, 84]	[33, 54, 55, 79]
Ventral tegmental area		[54]
Retrosplenial cortex		[33]
Lentiform nucleus	[80]	
Claustrum	[40, 80, 83]	
PAG	[81, 84, 85]	
Nucleus raphe	[34]	
Midbrain	[83]	
Temporal/parietal cortex		
Temporal pole		[32, 33, 54, 55, 82]
Inferior parietal lobe	[34, 40, 79–81, 83]	[33]
Precuneus	[83]	[32, 82]
Middle temporal gyrus	[84]	[79]
Superior parietal lobule	[78, 83, 84]	[79]
Transverse temporal gyrus	[40, 84]	
Occipital cortex		
Lingual gyrus		[79]
Cuneus		[32]
Occipital gyrus		[81]
Retrosplenial cortex		[32]
Angular gyrus		[82]
Fusiform gyrus	[84]	
Middle occipital gyrus	[84]	
Cingulate gyrus		
PCC	[82]	[32, 33, 55, 82]
pMCC	[34]	[33]
Subgenual cingulate	[82]	[34]
aMCC	[32, 34, 40, 83]	

**Table 3:** mixed-related BOLD responses evoked by acupuncture stimulation.

Regions	(References no.)	
	Activations	Deactivations
Somatosensory system		
SI	[71, 75]	[75]
SII	[72, 75, 76]	
thalamus	[77]	[53]
Supermarginal	[76]	
putamen	[71]	[71]
brainstem	[77]	[77]
Motor system		
M1	[71, 75, 76]	[74]
cerebellum	[53, 72, 74]	
SMA	[75, 77]	
Sensory integration system		
insula	[53, 71, 75–77]	[71]
ACC		[77]
Special senses		
IFG	[75, 76]	
STG	[75]	[53, 71]
MTG		[53]
Frontal cortex		
vmPFC		[77]
Frontal Pole		[74]
Frontal operculum	[75]	
Medial orbital prefrontal cortex		[72]
Medial frontal gyrus		[74]
DLPFC/VLPFC	[75, 76]	[75]
DMPFC	[75]	
Paracentral lobule		[77]
Amygdala	[76]	[77]
Hippocampus	[76]	
Parahippocampus	[53]	
Lentiform nucleus		[71]
PAG		[76]
Temporal/parietal cortex		
Inferior temporal gyrus		[72, 74]
Inferior parietal lobe	[72]	[75]
Precuneus		[71, 74, 75, 77]
Superior parietal lobule	[75]	
traisverse temporal gyrus	[72]	
Occipital cortex		
Lingual gyrus		[53, 75]
Cuneus	[75]	
Occipital gyrus		[53, 72, 74]
angular gyrus	[75]	
fusiform gyrus		[53]
Cingulate gyrus		
PCC		[71, 75, 77]
pMCC	[76, 77]	

**Table 4:** BOLD responses evoked by deep pain stimulation.

	Henderson et al., 2006 [37]	Henderson et al., 2008 [48]	Macefield et al., 2007 [87]	Niddam et al., 2002 [50]	Maeda et al., 2011 [88]
anterior cingulate		↑	↑	↑	↑
posterior cingulate		↑	↑	↑	↑
mid-cingulate	↑				
cingulate motor area (CMA)	↑				
primary somatosensory (S1)	↑	↑	↑	↑	
secondary somatosensory (SII)		↑	↑	↑	↑
motor cortices (M1)	↑			↑	
anterior insular	↑		↑	↑	
mid-insular	↑	↑			↑
posterior insular	↑		↑	↑	
cerebellar cortices		↑↓	↑		
Inferior frontal				↑	
Medial frontal				↑	
Middle frontal				↑	
Superior temporal				↑	
Inferior parietal				↑	↑
Claustrum				↑	↑
Thalamus				↑	↑
Precuneus				↑	
putamen					↑
caudate					↑
prefrontal (DLPFC)		↓			↑
perigenual cingulate (PACC)	↓	↓	↓		
hippocampus		↓			

**Table 5:** Methodological details of fMRI studies on BOLD responses for *de-qi*.

Studies	One group analysis method	*P* value of a one group analysis	Two group analysis method	*P* value of a two group analysis
Studies that compared central BOLD responses between *de*-*qi* and sharp pain groups				

Hui et al., 2000 [54]	Kolmogorov-Smirnov test	*P* < 0.001 or *P* < 0.00001 (cor)		
Hui et al., 2005 [33]	group-averaged	*P* < 0.001 (unc), minimum 3 voxels		
Hui et al., 2009 [55]	group-averaged	*P* < 0.001 (unc), minimum 3 voxels	two-sample unpaired *t*-tests	*P* < 0.05 (unc)
Sun et al., 2012 [40]	random effect model	*P* < 0.0001 (unc), minimum 5 voxels	two-sample unpaired *t-*tests	*P* < 0.0001 (unc)

Studies where no subjects reported sharp pain/excluded subjects who experienced sharp pain from fMRI analysis				

Napadow et al., 2005 [34]	Fixed effect model	*P* < 0.0001 (unc), minimum 3 voxels		
Jeun et al., 2005 [78]	random effect model	*P* < 0.01 (unc),		
Kong et al., 2007 [79]	random effect model	*P* < 0.05 (cor), minimum 10 voxels		
Zhou and Jia 2008 [66]	random effect model	*P* < 0.001 (cor) 50 voxels		
Na et al., 2009 [80]	random effect model	*P* < 0.001 (unc), minimum 100 voxels		
Bai et al., 2010 [81]	random effect model	*P* < 0.005 (unc), minimum 5 voxels		
Qiu et al., 2010 [82]	random effect model	*P* < 0.001 (unc), minimum 3 voxels		
Fang et al., 2012 [85]	random effect model	*P* < 0.05 (cor), minimum 5 voxels		
Sun et al., 2012 [40]	random effect model	*P* < 0.0001 (unc), minimum 5 voxels		
